# Optical Characteristics of ZnS Passivated CdSe/CdS Quantum Dots for High Photostability and Lasing

**DOI:** 10.1038/s41598-018-35768-8

**Published:** 2018-11-23

**Authors:** Xiongbin Wang, Jiahao Yu, Rui Chen

**Affiliations:** Department of Electrical and Electronic Engineering, Southern University of Science and Technology, Shenzhen, Guangdong, 518055 P. R. China

## Abstract

Nanocrystal quantum dots (QDs) have great potential for optoelectronic applications such as light emitting diodes and lasers due to their superior optical properties. The core-shell CdSe/CdS QDs can suppress Auger recombination effectively and enhance the emission efficiency. However, it will lead to poor photostability due to the small conduction band offset between CdSe core and CdS shell. For effective electron confinement, a thin shell of wide band gap ZnS semiconductor was grown on the CdSe/CdS core-shell QDs. The influence of ZnS shell has been investigated by comprehensive spectroscopic characterization. It is demonstrated that the CdSe/CdS/ZnS QDs show high photostable and temperature-insensitive emission. Moreover, room temperature lasing based on CdSe/CdS/ZnS QDs coated on a fiber was achieved. The lasing action can maintain under higher temperature up to 312.6 K. The experimental result is important for high performance optoelectronic device application based on colloidal QDs.

## Introduction

Colloidal semiconductor nanocrystals (NCs), or quantum dots (QDs), exhibit numerous advantages as light emitting materials, owing to their tunable emission, solution processability, and high photoluminescence (PL) quantum yield (QY)^1–3^. All these unique properties make them suitable for potential application in the third-generation photovoltaics, light emitting diodes (LEDs) and lasers^1,3–5^. Nevertheless, all these applications have been severely limited not only by the Auger recombination (AR), a nonradioactive process during which an electron-hole pair transfers its energy to a third carrier, but also the photostability and surface defects that could result in non-radiative recombination^6^. Various methods have been proposed and demonstrated to solve these problems, and it is widely accepted that heterogeneous material epitaxy is the most effective one. When choosing materials for hetero-epitaxy, many things should be taken into consideration such as lattice constant, energy gap and band offset^7,8^. For CdSe QDs, CdS is widely accepted as a better choice for hetero-epitaxy growth. Klimov *et al*. first demonstrates that the AR in CdSe could be efficiently suppressed by coating the QDs with a CdS shell, in which the core-shell structure was known as type-II or quasi-type-II quantum structures^9–16^. In the type-II quantum structures, the hole and electron is confined at different regions, which could decrease the overlapping of the electrons and holes wavefunction. Such weaker interaction will result in the suppression of AR^9^.

A thin shell of a wide band gap semiconductor grown on the emitting QDs could substantially improve their stability^17^. In case of CdSe/CdS, the lattice mismatch between the core and shell is relatively small which gives rise to the good crystal quality of the materials. As a result, the CdSe/CdS QDs always possess high PL QY than bare QDs^18–20^. However, the small band offset between CdSe and CdS could not provide effective confinement for electrons. In the case of CdSe/ZnS, the ZnS shell would be a more suitable passivation layer for CdSe core owing to its large band gap (3.8 eV for bulk material). Nevertheless, the large lattice mismatch will create dangling bonds or strain at the interfaces which negatively affect both the PL QY and the photostability^21,22^. To solve the problems, a ZnS shell grown on the CdSe/CdS QDs provides efficient confinement of electrons and holes inside the QDs as well as highly photostability. Meanwhile, the middle shell (CdS) allows the suppression of strain due to small lattice mismatch between the CdS core and ZnS shell. In addition, the ZnS shell could absorb high energy photons and then carriers will diffuse into CdSe core, which contributes to the high emission efficiency.

Here, optical characteristics of CdSe/CdS/ZnS QDs were investigated through comprehensive spectroscopic study. Photostable and temperature-insensitive QDs have been obtained by analyzing the influence of ZnS shell on CdSe/CdS QDs. Thanks to the improved optical properties of QDs, laser action is successfully obtained based on whispering gallery mode (WGM) at room temperature by coating the QDs on the fiber surface. Furthermore, lasing at higher temperatures up to 312.6 K was demonstrated, which indicates the superior photostability of the samples.

## Results

**Figure 1:**
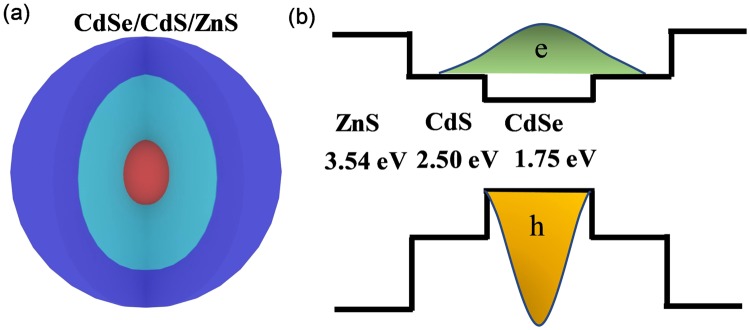
(**a**) Schematic diagram of CdSe/CdS/ZnS nanostructure. (**b**) Band structure of the material.

The schematic CdSe/CdS/ZnS core-multi-shell structure is shown in Fig. 1(a). The energy band structure is one of the most crucial factors to determine the optical characteristics of the semiconductors QDs. As shown in the band alignment diagram in Fig. 1(b), CdSe and CdS is characterized by a relative small conduction band offset that electrons could migrate from the CdSe core to the CdS shell, while holes are totally confined at the CdSe core region due to the large valance band offset. Comparing with CdSe/CdS energy band structure, both the valance and conduction band offset between CdS and ZnS is larger, which indicate that electrons and holes shall be well-confined in the same region of the core-multi-shell QDs.

**Figure 2:**
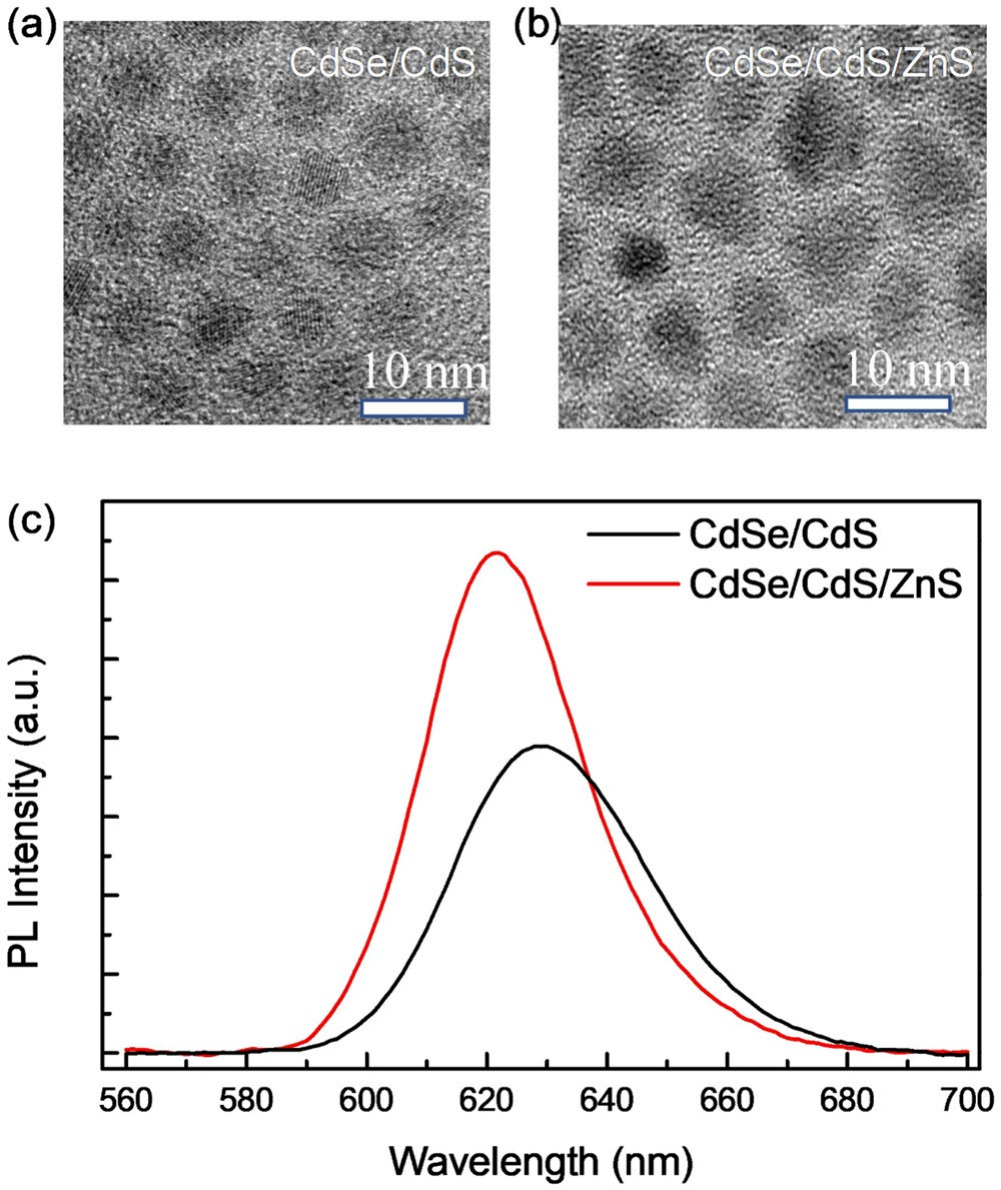
High resolution TEM image of (**a**) CdSe/CdS and (**b**) CdSe/CdS/ZnS QDs. The correspond scale bar is 10 nm. (**c**) Room temperature PL spectrum of CdSe/CdS and CdSe/CdS/ZnS QDs in solution.

Steady-state morphology characterizations and PL measurements were carried out to verify the successful growth of ZnS shell upon the CdSe/CdS core-shell structures. It is expected that the electronic structure and the corresponding optical property will change with the additional ZnS shell. Figure 2(c) shows the room temperature PL spectrum of the reference CdSe/CdS and the CdSe/CdS/ZnS QDs in solution (The PL intensity is not comparable owing to different concentration of our samples). The PL spectrum reveals a blue shift from CdSe/CdS/ZnS QDs (which is around 621 nm) compared with the emission of CdSe/CdS QDs (which is around 631 nm). The blue shift is mainly due to the partial formation of a Cd_x_Zn_1-x_S alloy shell. That is because the temperature during the material growth is relatively high, so that the Zn-atoms will diffuse into the Cd rich regions of the shell, thus increasing the band-offset of the shell and hence the effective confinement^23,24^. The blue shift also indicates the successful synthesis of the ZnS shell. The absolute PLQY of CdSe/CdS and CdSe/CdS/ZnS QDs are measured to be around 0.257 and 0.412, respectively. The higher absolute PLQY shows that the ZnS shell could provide good passivation of the CdSe/CdS QDs. The shell formation could also be evidenced from the high-resolution transmission electron microscopy (TEM). According to the TEM images (Fig. 2(a) and (b)) and the size distribution, the QDs are about 4.96 ± 0.34 nm and 6.57 ± 0.70 nm, respectively. It is important to note that the average size of the CdSe/CdS/ZnS QDs is larger than the CdSe/CdS QDs, which supports the successful growth of the ZnS shell. Comparing the size of CdSe/CdS/ZnS QDs with the as-grown sample, the former one shows better dispersity.

**Figure 3:**
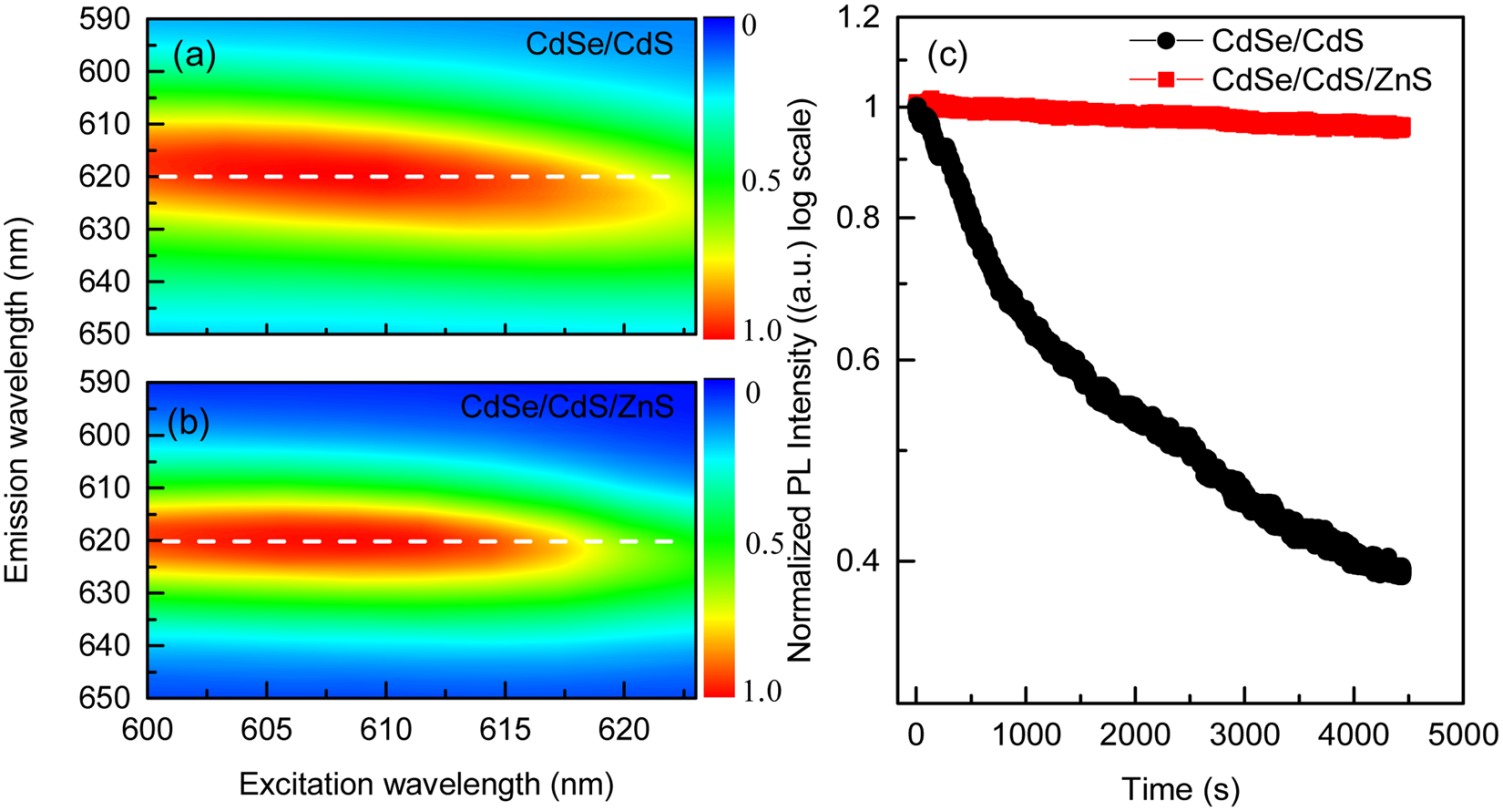
Two-dimension contour plot of emission wavelength verses excitation wavelength for (**a**) CdSe/CdS QDs and (**b**) CdSe/CdS/ZnS QDs. The color represents the normalized intensity of emission. (**c**) Normalized PL intensity (in log scale) of the samples with time.

Generally, the probability of a resonant transition between the excited state and the ground state will be much greater than the probability of other transitions. The band gap of QDs is related not only to the energy gap of the bulk material, but also their size. To characterize the optical property of the materials, a resonant PL measurement is applied to the two sorts of samples (Fig. 3(a) and (b)). During the measurement, the excitation wavelength is varying from 600 nm to 625 nm. Under resonant excitation, the emission peak of CdSe/CdS QDs shows an obvious red shift with the excitation wavelength (Fig. 3(a), the red region demonstrates a deviation from the white dash line). In contrast, there is no distinct deviation of the emission peak for the CdSe/CdS/ZnS QDs, which could be attributed the good size-homogeneity (Fig. 3(b)) consisted with the observation of QDs from the TEM image. It has been reported that the photostability of the QDs could be enhanced with the thickness of the outer shell, which can be ascribed to the surface modification that the shell separates the core from environment^25^. A stability testing of QDs film is performed to prove the improved photostability. As can be seen in Fig. 3(c), the PL intensity of CdSe/CdS QDs decreases dramatically, while it is greatly improved for CdSe/CdS/ZnS QDs film under identical excitation.

**Figure 4:**
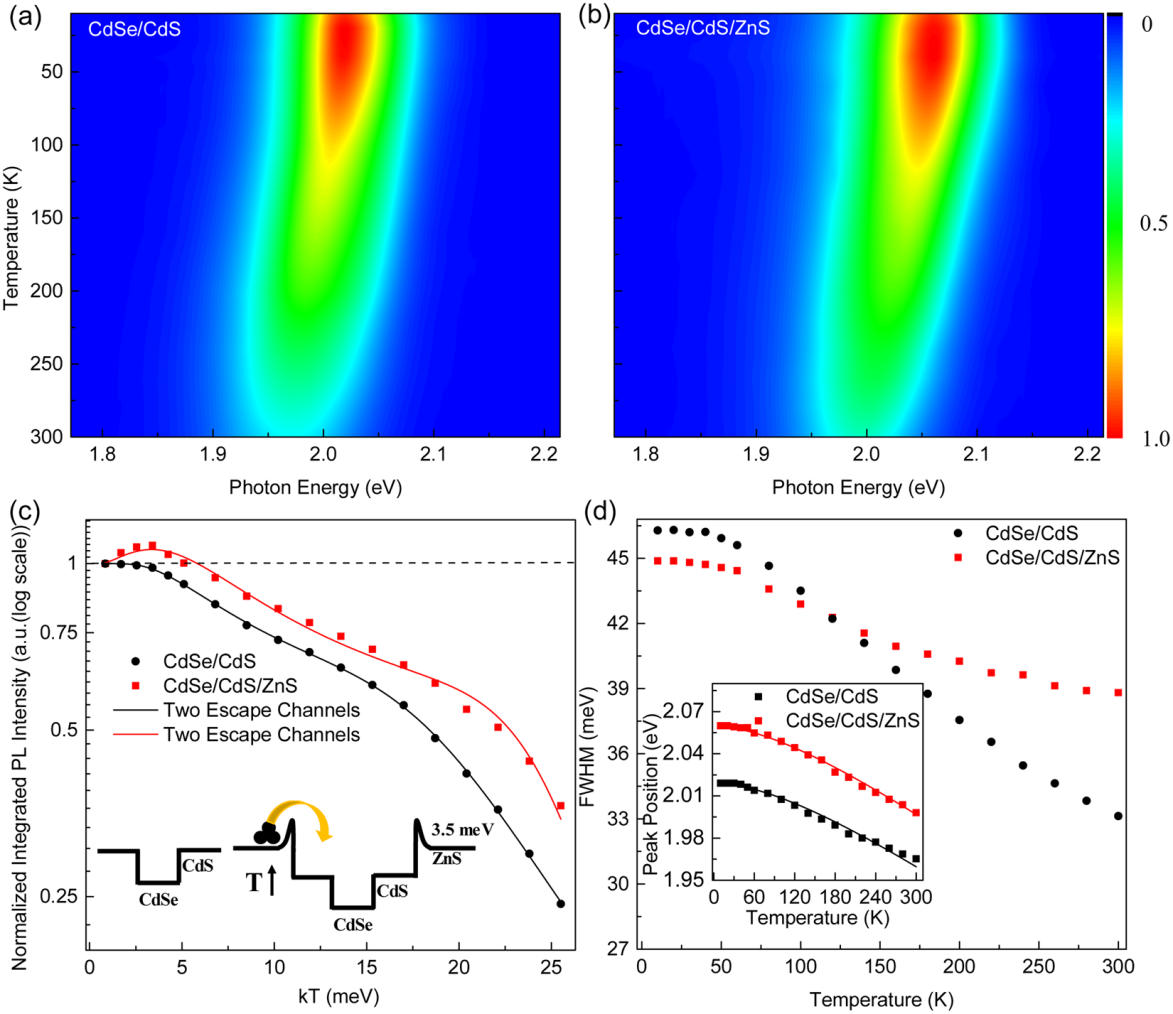
Temperature dependent PL measurement of (**a**) CdSe/CdS and (**b**) CdSe/CdS/ZnS QDs from 10 to 300 K. (**c**) The integrated PL intensity (normalized) of the samples with temperature. The data is fitted by equation (2). The inset shows that trapped carrier could overcome the potential barrier with increasing temperature. (**d**) The band width of the samples at different temperatures. The inset shows the peak positions with temperature, and was fitted by Varshni formula.

To discuss the optical characteristics of the materials, temperature dependent PL measurement is applied to investigate how the ZnS shell affects the PL behaviors of CdSe/CdS core shell QDs. Temperature dependent PL emission from 10 to 300 K of the two samples is shown in Fig. 4(a) and (b). Figure 4(c) shows the normalized integrated PL intensity of samples with temperature. It can be seen that the normalized integrated PL intensity of CdSe/CdS/ZnS QDs first increases and reach a maximum, then decreases with temperature, while the normalized integrated PL intensity of CdSe/CdS shows a monotonous decrease. Quantitatively, the temperature dependent PL intensity could be described by using the model proposed by Popescu *et al*.^26^. The integrated PL intensity at low temperature could be express as

1 $$\frac{{R}_{r}{N}_{D}}{{G}_{e}}=\frac{1}{1+\frac{{K}_{0}}{1+\exp (-\frac{{E}_{b}}{kT})}}$$

where *R*_*r*_ is the electron-hole pairs recombination rate of QDs, and *N*_*D*_ is the population of electrons in the QDs. *G*_*e*_ is the number of carriers captured by the QW per unit time. *K*_0_ is a constant, *k* is the Boltzmann constant, *T* represents temperature, and *E*_*b*_ shows the potential barrier. For temperature at high range, thermal escaped carriers should be considered, and the equation could be amended as follow

2 $$\frac{{R}_{r}{N}_{D}}{{G}_{e}}={\{1+\frac{{K}_{0}}{1+\exp (-\frac{{E}_{b}}{kT})}[1+\sum _{i=1}{K}_{i}exp(-\frac{{E}_{i}}{kT})]\}}^{-1}(i=1\,or\,2)$$

where *i* is the number of thermal escape channels for carriers, *K*_*i*_ is a constant, and *E*_*i*_ represents the thermal activation energy.

By fitting the experimental data at low temperature range (10–40 K) using Eq. (1), the value of the potential barrier of *E*_*b*_ for CdSe/CdS/ZnS is estimated to be 3.5 meV. The potential barrier locates at the interface of CdS and ZnS could trap the carriers at low temperature. As the temperature increase, trapped carriers will gain enough thermal energy to overcome this potential barrier, relax into the CdSe/CdS region, and then contribute to the emission of the sample (as shown in the inset of Fig. 4(c)). Later on, Eq. (2) was used to fit the experimental data through the whole temperature range for both QDs. Initially, one channel is used to fit the experiment data but it could only fulfils the data lower than 220 K. It indicates that more channels for thermal escape should be considered. The temperature dependence of QDs integrated PL intensity could be perfectly fitted when two thermal escape channels are considered. For CdSe/CdS core shell QDs, the activation energy is obtained around to be 14 and 300 meV, respectively. The 14 meV activation energy is attributed to the thermal activated defects that could capture the exciton and release the energy through a nonradiative pathway. The 300 meV activation energy is close to conduction band offset between CdSe and CdS. For the CdSe/CdS/ZnS QDs, the two activation energies obtained are 18 and 300 meV, respectively. Similar to CdSe/CdS QDs, the 300 meV represents the energy difference of conduction band between CdSe and CdS. The 18 meV activation energy could also be considered to the thermal activated nonradiative defects at the interface of ZnS shell and CdS shell. Bearing in mind, the relative larger activation energy 18 meV comparing with 14 meV which share the same origin shows that the ZnS could suppress the defect at the surface. Furthermore, it can be seen that the PL intensity of CdSe/CdS QDs decreases rapidly with increasing temperature, and it remains only 24% at room temperature compared to its initial (10 K) emission. In contrast, CdSe/CdS/ZnS QDs are able to maintain 35% of the initial PL intensity, which indicates that an additional ZnS shell could improve the temperature stability of QDs.

Apart from the normalized PL intensity, the full width half maximum (FWHM) information (Fig. 4(b)) and peak position (inset in Fig. 4(d)) were extracted for discussion from our experimental data. The FWHM of CdSe/CdS QDs show a dramatically decrease with temperature while the CdSe/CdS/ZnS QDs demonstrate a slower trend. The different tendency may be attributed to the strain near the interface or the existence of localized states. As for the peak position, Varshni equation is used to describe the tendency^27,28^. The Varshni relation could be express as

3 $${E}_{g}(T)={E}_{g}(0)-\,\frac{\alpha {T}^{2}}{T+\beta }$$

where *E*_*g*_(0) is the band gap at 0 K, *α* is the temperature coefficient, and *β* is a parameter related to the Debye temperature. For CdSe/CdS QDs, *α* = 3.55 × 10^−4^ *eV*/*K*, *β* = 240 ± 30 *K*. For CdSe/CdS/ZnS QDs, *α* = 3.65 × 10^−4^ *eV*/*K*, *β* = 225 ± 30 *K*. The obtained parameters are close to those CdSe bulk and nanodots^29,30^.

**Figure 5:**
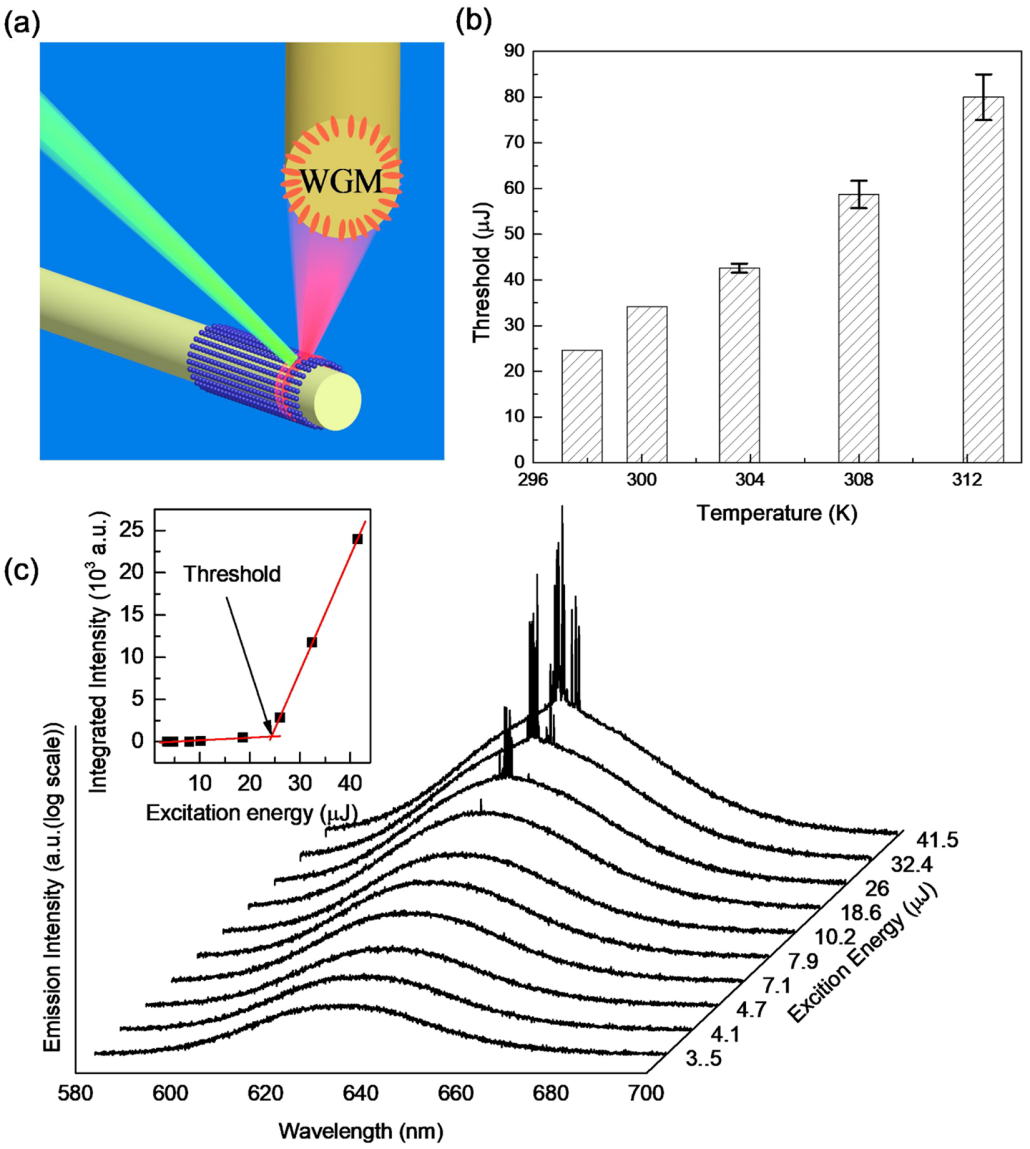
(**a**) Schematic diagram of micro-laser by coating QDs on fiber. (**b**) Pump intensity-dependent emission (in logarithmic scale) of CdSe/CdS/ZnS QDs at room temperature. The inset represents narrowing integrated PL intensity of peaks (solid squares). (**c**) Thresholds of the lasing from QDs at different temperatures.

Lasing has been successfully obtained based on CdSe/CdS/ZnS QDs with a spherical optical cavity at room temperature. The QDs are coated on a fiber which can be served as a circular cavity. The emitted light propagates around the circumference owing to total internal reflection. The resulting model could be regard as whispering galley mode (WGM). As shown in Fig. 5(a), the QDs are excited by a pulse nanosecond laser at 532 nm. At low pumping region, only a broad spontaneous emission peak is observed. When the pump energy increases to certain value, some sharp peak with equally space appear which could be attributed to the lasing behavior from the WGM at room temperature (Fig. 5(b)). Notice that the y-ordinate in Fig. 5(b) is logarithmic scale. The graph of integrated PL intensity shows a transition from linear to a nonlinear tendency, implying the development of lasing action with a threshold around 24.5 μJ/pulse (Fig. 5(a) inset). Pump intensity dependent measurement is carried out at different temperatures. Lasing behavior can be successfully obtained with temperature increasing to 312.6 K which could be ascribed to the great enhancement by ZnS shell. The thresholds of lasing at different temperatures are estimated, rising from 24.5 μJ/pulse to 67.2 μJ/pulse at 312.6 K (Fig. 5(c)).

## Discussion

In conclusion, optical characteristics of CdSe/CdS/ZnS QDs were investigated through a comprehensive steady-state spectroscopic study. By analyzing the influence of ZnS shell on CdSe/CdS QDs, photostable and temperature-insensitive QDs have been obtained. Thanks to the improved optical properties of QDs, laser action is successfully obtained based on whispering gallery mode (WGM) at room temperature by using QDs as optical gain media. Furthermore, lasing at higher temperatures up to 312.6 K was demonstrated, which results from the superior photostability of the samples. Our result shows that CdSe/CdS/ZnS QDs have great potential to serve as laser material for better temperature stability and higher temperature operation.

## Methods

### Synthesis of QDs

The core-shell and core-multi-shell QDs were synthesized according the procedure described in the reported literature^31^. In short, 1 mmol cadmium oxide, 18.85 mmol oleic acid zinc acetate were mixed in a flask with 25 mL 1-octadecene. Then, selenium precursor in trioctylphosphine (TOP, 0.2 mmol) was added into the flask. After that, dodecanethiol was injected into the mixture. Then, CdSe/CdS QDs were obtained. Subsequently, sulfur precursor in TOP with a concentration of 1 mmol was injected into the flask to obtain CdSe/CdS/ZnS QDs. Finally, the QDs were purified and re-dissolved in toluene.

### Laser Spectroscopy

Absolute PLQY was measured by Hamamatsu Quantaurus-QY Absolute PL Quantum Yield Spectrometer in hexane excited at 500 nm. For continuous wave excitation, 532 nm was selected from a Xenon lamp combines with a monochromator. For the stability testing of the QDs film, the power of the light is fixed at 0.2 mW. The temperature dependent PL measurements of the samples were performed between 10 and 300 K within a helium closed-cycle cryostat, and the signal was dispersed by a 750 mm monochromator combined with suitable filters, and detected by a photomultiplier using the standard lock-in amplifier technique. For high density excitation, the laser source was replaced by a frequency-doubled, Q-switched Nd:YAG laser which produced green pulse at the wavelength of 532 nm. The signal was detected by a UV-enhanced charged coupled device (CCD). The pulse width and repetition rate of the laser are about 1 ns and 60 Hz, respectively.
